# Antimicrobial activity of silymarin mediated zinc oxide and hydroxy apatite nanoparticles against oral pathogens

**DOI:** 10.6026/97320630016863

**Published:** 2020-11-30

**Authors:** S Aravind Kumar, s Rajeshkumar, SP Saravana Dinesh, Ashwin Mathew George, Ravindra Kumar Jain

**Affiliations:** 1 Department of Orthodontics, Saveetha Dental College and Hospital, Saveetha Institute of Medical and Technical Science (SIMATS), Saveetha University, Chennai - 600077, Tamilnadu, India; 2 Nanobiomedicine Lab, Department of Pharmacology, Saveetha Dental College and Hospital, Saveetha Institute of Medical and Technical Science (SIMATS), Saveetha University, Chennai - 600077, Tamilnadu, India

**Keywords:** Hydroxyapatite nanoparticles, green synthesis, silymarin, zinc oxide nanoparticles, oral pathogens

## Abstract

The nanoparticles such as hydroxyapatite, zinc oxide, titanium dioxide and zirconium nanoparticles have application in dentistry. Therefore, it is of interest to document the antimicrobial activity of silymarin mediated zinc oxide and hydroxy apatite nanoparticles against oral pathogens. Hence, we synthesized hydroxyapatie and zinc oxide nanoparticles with silymarin and characterized by UV-visible spectrophotometer. Data shows that silymarin mediated HAP and ZnO nanoparticles have antimicrobial activity against oral pathogens such as Pseudomonas sp, Staphylococcus aureus, Streptococcus mutans, Enterococcus faecalis and Candida albicans.

## Background

Nanotechnology is employed to treat microbial infections [1]. Nanoparticles are incorporated in antibacterial coatings for implants in biomaterials to prevent bacterial infection and promote wound healing. It is also used in microbial diagnosis [2,3]. Nano materials due to its particle size, larger surface area, increased chemical reactivity, proven to be broad-spectrum antimicrobial agent for treating dental infections caused by bacterial accumulation in the oral cavity [4]. Nanoparticles are used in various fields of dentistry including endodontics, prosthodontics, periodontics and orthodontics. Dental plaque/biofilm accumulation is commonly seen in orthodontic appliances/treatments. This will enhance the chance of enamel demineralization, which is commonly called as white spot lesions caused due to organic acid produced by the microbial biofilms [5- 6]. This can be managed by providing oral hygiene education. Moreover, mechanical therapy can prevent and remove the plaque biofilm. However, more effective methods are employed to treat the plaque formation. Natural products/ herbal based compounds are proven to be effective treatment due to their antimicrobial, antioxidant and anti-inflammatory effects. Nonetheless, the exact mechanism of action is still unknown. Addition of antimicrobial nanoparticles to orthodontic adhesives and resin modified glass inomeric cements prevents the invading microbes in the oral activity [7]. It is known that hydroxyapatite nano fillers had good antimicrobial property and shear bond strength [8]. Zinc oxide nanoparticles used in the field of biomedicine for their physicochemical properties is popular due to its UV absorption properties [9-10]. It shows significant antimicrobial, antioxidant and wound healing efficacy. It is cost effective with low toxicity metal oxide [11]. The antibacterial and anti cancer effects are exhibited by activation of apoptotic signalling pathway, destroying the membrane integrity and Intracellular through reactive oxygen species [12-13]. Silymarin derived from milk thistle Silybum marianum is a known hepato protective agent [14]. It has been shown to possess various pharmacological activities like hepato protective, antioxidant, anti inflammatory, anticancer, and cardio protective effects [15]. Silymarin is primarily used in management of periodontal infections and oral cancer. It is effective antimicrobial drug to treat dental cariesdental plaque. It also inhibits the growth of oral bacteria either alone or in combination with other antibiotics [16,17, 18]. Therefore, it is of interest to document the antimicrobial activity of silymarin mediated zinc oxide and hydroxy apatite nanoparticles against oral pathogens such as Streptococcus mutans, Lactobacillus acidophilus, E. faecalis.

## Materials and Methods:

### Preparation of silymarin extract:

0.5g of silymarin powder was measured and dissolved in 100mL distilled water and boiled for 10minutes at 60-80°C using a heating mantle. The boiled mixture was filtered using Whatmann No.1 filter paper. The filtered extract was stored in refrigerator.

### Preparation of zinc oxide nanoparticles (silymarin)

20 millimolar Zinc acetate was measured (0.876g) and dissolved in 60 ml of distilled water. To that 40ml of filtered silymarin extract was added. To obtain uniform dispersion the prepared solution was maintained in an orbital shaker at 120rpm for 3-4 days. To confirm the synthesis of zinc nanoparticles UV Visible spectrophotometry was taken at regular specific intervals in the wavelength range of 250-650nm.

### Preparation of hydroxy apatite nanoparticles (silymarin)

10millimolar Hydroxy apatite (0.502g) was measured and dissolved in 60ml of distilled water .To that 40ml of filtered silymarin extract was added. To obtain uniform dispersion the prepared solution was maintained in an orbital shaker at 120 rpm for 3-4 days. To confirm the synthesis of HAP nanoparticles UV Visible spectrophotometry was taken at regular specific intervals in the wavelength range of 250-650 nm.

### Centrifugation:

The synthesized nanoparticle solution was centrifuged at 8000rpm for 10 mins. After centrifugation, the supernatant was discarded and the pellet was stored.

### Antimicrobial activity (agar well diffusion technique):

Mueller Hinton Agar was prepared, and sterilized using autoclave at 121°C for 15-20minutes. The sterile MHA media was poured on the surface of the sterile Petri plates and allowed for solidification. After solidification, the organisms such as Pseudomonas aeruginosa Staphylococcus aureus, Streptococcus mutans, Enterococcus faecalis were swabbed using sterile cotton buds. The wells were made using a T – shaped well cutter. Among four wells per plate 3 wells were loaded with silymarin zinc acetate pellet solution in the concentration range of 25µL, 50µL, 100µL and the fourth well loaded with a standard antibiotic (amoxyrite). Then the plates were incubated at 37°C for 24 hours. After incubation, the plates were observed and measured for Zone of inhibition around the nanoparticle loaded wells. Rose Bengal Agar was prepared as the medium for C. albicans and the inoculated plates were incubated at 37°C for 48 hours.

## Results and Discussion:

### Visual observation:

**Figure 1:**
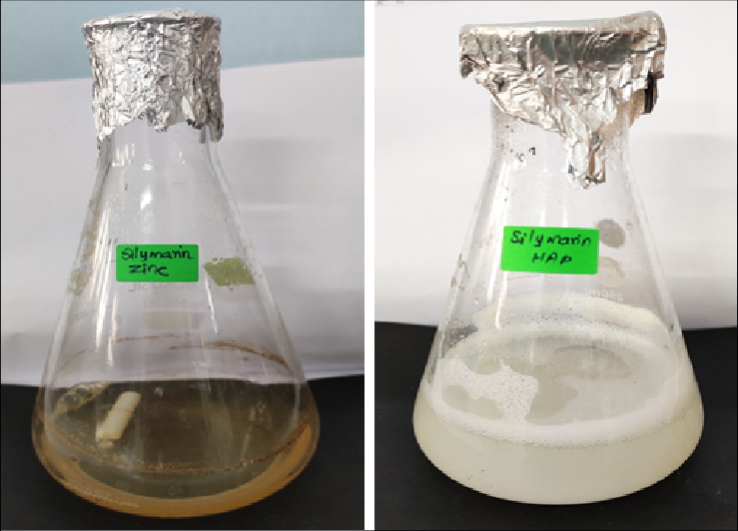
Silymarin mediated zinc oxide and HAP nanoparticles

Figure 1 shows the green synthesis of hydroxyapatite and zinc oxide nanoparticles. The silymarin reacts with the hydroxyapatite and zinc acetae and forming the nanoparticles. When compared with initial addition in the 24 h the formation cloudiness and slight modoifcations in the solution indicates the nanoparticles formation [19,20].

### UV-visible spectroscopy:

**Figure 2:**
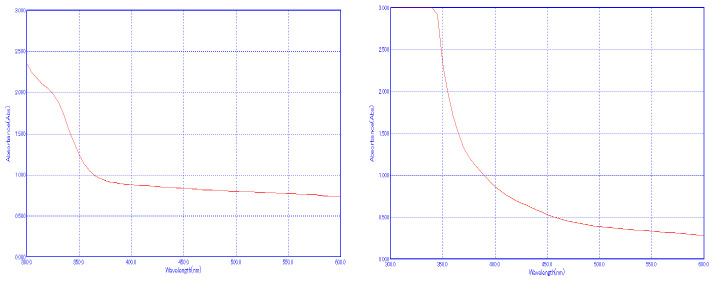
UV-visible spectroscopic analysis of silymarin mediated HAP and ZnO nanoparticles

The broad peak at 540 nm confirms the synthesis of hydroxyapatite nanoparticles and peak at 340 nm confirms the zinc oxide nanoparticles using silymarin. Figure 2 clearly shows the surface plasmon resonance of nano Hap and ZnO nanoparticles. The zinc oxide nanoparticles synrhesized using different plant extracts also confirms the nanoparticles synthesis [21,22].

### Antimicrobial activity:

**Figure 3:**
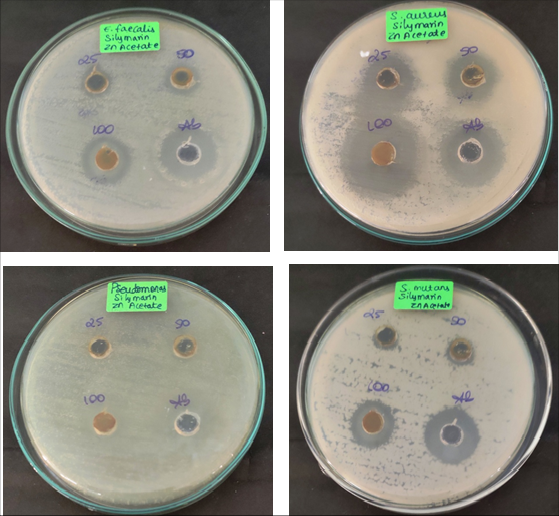
Antibacterial activity of HAP nanoparticles

The Figure 3-Figure 6 shows the antibacterial activity of HAP nanoparticles against pseudomonas sp, S. aureus, S. mutans and E. faecalis in that the HAP nanoparticles shows zone of inhibition against S. mutans.

**Figure 4:**
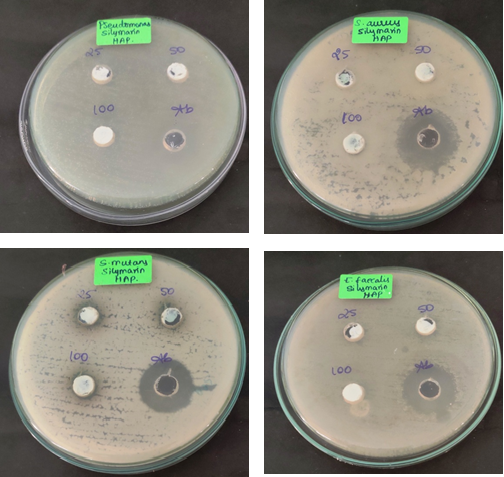
Antibacterial activity of zinc oxide nanoparticles

**Figure 5:**
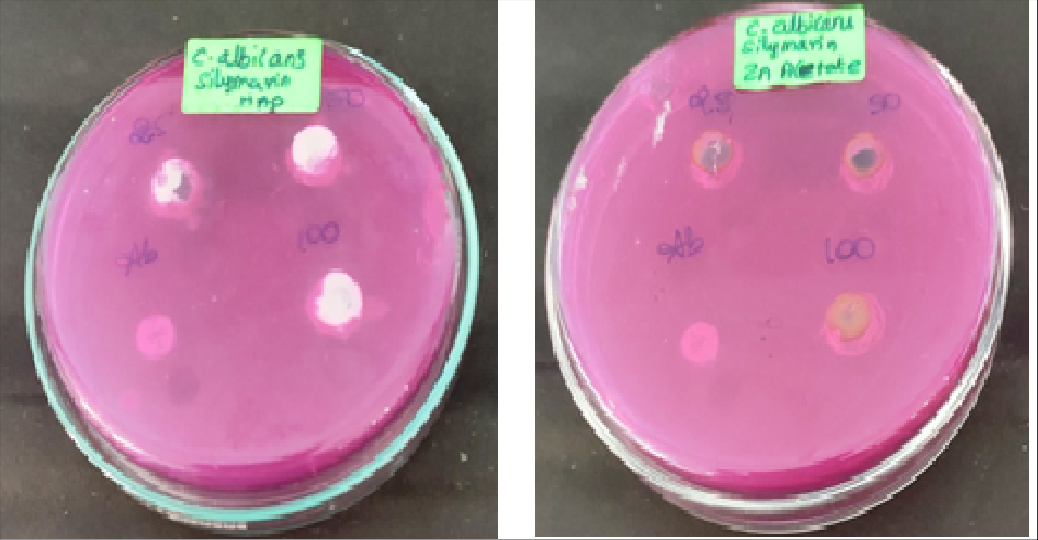
Antifungal activity of HAP and zinc oxide nanoparticles

**Figure 6:**
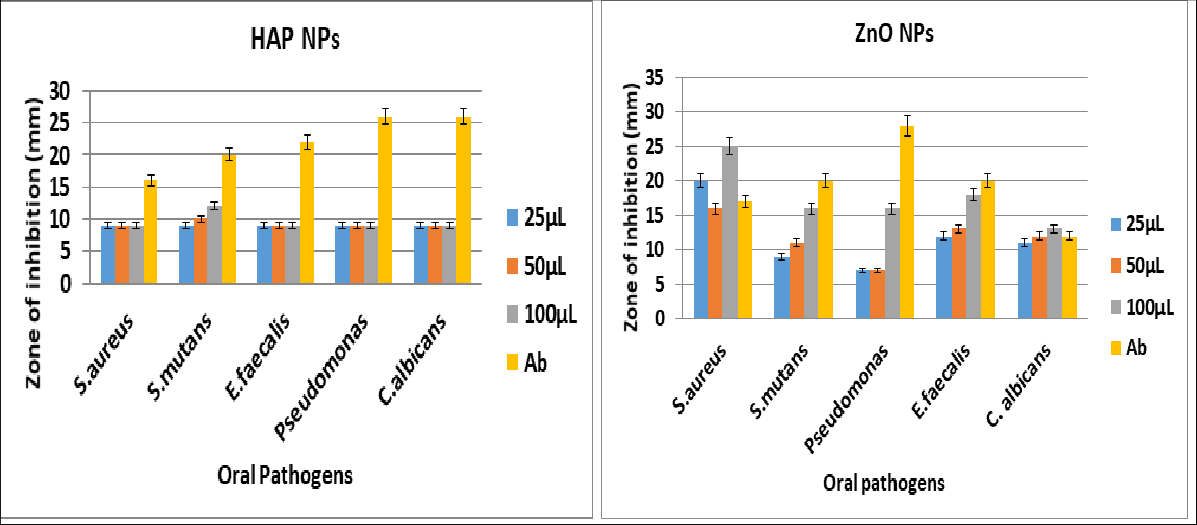
Antimicrobial activity of HAP and zinc oxide nanoparticles

The zinc oxide nanoparticles shows very good antibacterial and antifungal activity against all oral pathogens such as pseudomonas sp, S. aureus, S. mutans, C. albicans and E. faecalis. In that the zinc oxide nanoparticles shows more activity when compared with standard drug. Figure 4, Figure 5, and Figure 6 cleary the shows the antimicrobial activity of silymarin mediated zinc oxide nanoparticles. The zinc oxide nanoparticles synthesized using different plant extract shows good antibacterial, antifungal and anticancer activity [23,24,25,26].

## Conclusion

The application of nanoparticles in dentistry is gaining momentum. Results show that silymarin mediated HAP and ZnO nanoparticles have antimicrobial activity against oral pathogens such as Pseudomonas sp, Staphylococcus aureus, Streptococcus mutans, Enterococcus faecalis and Candida albicans.
